# Management of periprosthetic proximal femoral fracture of megaprosthesis with limited residual bone stock: A case report

**DOI:** 10.1016/j.ijscr.2025.112138

**Published:** 2025-11-05

**Authors:** Muhamad Naseh Sajadi Budi, Bangkit Primayudha, Mohammad Syarif Mas'ud, Greesea Dinamaria Whitiana, Andre Prawiradinata

**Affiliations:** a Department of Orthopaedics, Padjadjaran University, Hasan Sadikin Hospital, Bandung, Indonesia

**Keywords:** Periprosthetic fracture, fibular strut graft, limited bone stock

## Abstract

**Introduction:**

Periprosthetic fractures involving femoral megaprostheses are complex and challenging to manage due to limited residual bone stock available for fixation. In Indonesia, data on such complications are scarce, and surgical strategies often require adaptation based on resource availability.

**Case presentation:**

A 42-year-old man with a history of distal femoral megaprosthesis implantation for a giant cell tumor presented with right hip pain after a low-energy fall. Imaging showed a Vancouver type B3 periprosthetic fracture. The remaining proximal femoral bone stock measured only 7.5 cm, significantly limiting options for internal fixation due to poor surface area and compromised load distribution. Surgical management involved internal fixation using a trochanteric claw plate and cable wires. Dual 8 cm autologous fibular strut grafts were harvested and positioned anteriorly and posteriorly around the fracture site. Postoperative recovery was uneventful. At four-month follow-up, the patient demonstrated excellent progress, with no signs of infection, good range of motion, and absence of pain.

**Discussion:**

This case illustrates the viability of using a trochanteric claw plate and fibular strut grafts to treat periprosthetic fractures around a stable femoral megaprosthesis. The fracture likely occurred due to bone fragility at the cement–bone interface. The combination of mechanical fixation and biological grafting enabled early mobilization while preserving the existing implant.

**Conclusion:**

In resource-limited settings, this technique offers a promising limb-salvage strategy with favorable short-term outcomes. Further studies are needed to validate long-term effectiveness.

## Highlights

• This case demonstrates a successful limb-salvage technique for managing a periprosthetic femoral fracture around a stable megaprosthesis with severely limited residual bone stock.
• A combination of trochanteric claw plate fixation and dual autologous fibular strut grafts provided both mechanical stability and biological support.
• The approach is practical and reproducible, offering a viable solution for resource-limited settings with favorable short-term outcomes.

## 1 Introduction

The use of megaprostheses in managing bone tumors has become a cornerstone of modern limb-salvage surgery, particularly for malignant primary bone tumors such as osteosarcoma and chondrosarcoma [1,2]. In North America and Europe, 70–90% of primary bone sarcoma cases are now managed with limb-salvage procedures using modular megaprostheses, replacing amputations as the standard of care [3,4].

In Indonesia, however, the epidemiology and outcomes of megaprosthesis use remain sparsely documented and largely confined to single-center case series in major referral hospitals. These studies indicate that megaprosthesis procedures are relatively rare and are predominantly performed in tertiary centers in Jakarta and Surakarta. A notable case series conducted in Jakarta reviewed 32 patients underwent limb salvage surgery with megaprosthesis between 2011 and 2015 [5]. Another study from Surakarta analyzed 17 cases involving distal femur and proximal tibia megaprosthesis replacements for primary bone tumors, including osteosarcoma and giant cell tumors [6]. Overall, the utilization of megaprostheses in Indonesia is constrained by factors such as high costs, limited access to modular implants, and the absence of a centralized musculoskeletal tumor registry. These barriers sharpen the need for context-appropriate, resource-adapted solutions [5,6].

Despite oncologic and functional benefits, complications associated with megaprosthesis use in bone tumor reconstruction are significant and multifactorial. The most frequently reported issues include periprosthetic fractures, aseptic loosening, infection, mechanical failure, and extensor mechanism disruption [7]. Savvidou et al. highlighted that complication rates with megaprostheses are 5 to 10 times higher than those for standard total joint arthroplasties, with infection (34%) and structural failure (17%) being major contributors to implant failure [8]. Vaishya et al. documented a case of periprosthetic subtrochanteric fracture post-knee megaprosthesis, noting the challenges of fracture management near large implants, often requiring revision surgery or internal fixation [9]. Collectively, studies comparing megaprosthesis to ORIF in elderly patients with distal femur periprosthetic fractures, report a higher complication and revision rate in the megaprosthesis group (50%) versus the ORIF group (11.1%), primarily due to infection and wound healing issues, thus emphasizing that implant choice must balance biology, soft-tissue condition, and host factors [[10], [11], [12]].

Data on periprosthetic fractures following megaprosthesis implantation in Indonesia remain scarce, with most information derived from isolated case series and retrospective reports from tertiary centers, lacking comprehensive national registries or multicenter studies to quantify true incidence and outcomes. Within this constrained evidence base, especially scarce in Indonesia, we report a short-term follow-up of a periprosthetic proximal femoral fracture around a distal femur megaprosthesis, successfully managed using a trochanteric claw plate, cable wiring, and autologous fibular strut grafts. This resource-adapted strategy preserved a stable oncologic reconstruction where revision tumor components or custom augments may be unavailable or financially prohibitive, while biologic augmentation expanded healing surface and off-loaded the construct [5,6]. This report follows the SCARE 2025 reporting guideline [13].

## 2 Case description

A 42-year-old man came to emergency department with chief complaint of pain in the proximal right femur after he had fallen with a sitting position in his house one day prior to admission. There was no other pain in any other parts of the body nor radiation of the pain from the proximal right femur. He also complained that he lost the ability to bear his weight upon standing on both feet.

He had a history of right distal femur reconstruction using megaprosthesis due to Giant Cell Tumor in 2017 at the same hospital. The surgery went well as he was able to walk and do activity with no assistance. There was no fever nor wound discharge postoperatively. Radiotherapy and chemotherapy treatments were not given to the patient.

**Fig. 1:**
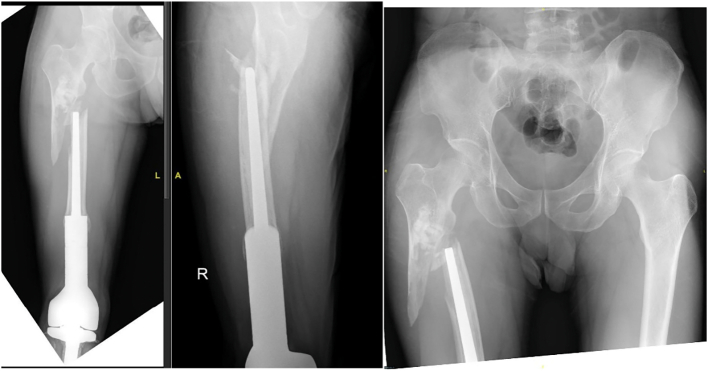
Plain radiograph showing periprosthetic fracture at the right proximal femur.

The physical examination found that the patient was fully alert with normal vital signs (blood pressure, heart rate, respiratory rate, body temperature, and oxygen saturation). Upon examination at the location of the pain, we found deformity and shortening, as well as swelling and redness. The leg length discrepancy was at 3 cm, the true length was 77 cm for right limb and 80 cm for left limb, and the apparent length was 83 cm for right limb and 86 cm for left limb. Patient had complaint of tenderness; no prolonged capillary refill time or disturbance in the distal sensibility compared to the left femoral region was found. Upon active and passive movement, the range of motion of the patient was limited. Femoral X-ray of the patient revealed a bone discontinuity at the right subtrochanteric femur. Radiographic assessment revealed a very limited residual bone stock proximal to the prosthesis, with only 7.5 cm of bone available for fixation (Fig. 1). The patient was diagnosed with periprosthetic fracture at right subtrochanteric femur Vancouver classification type B3 and ⁠post megaprosthesis total knee replacement due to Giant Cell Tumor at right knee joint. The patient was given analgesic and immobilized by skin traction.

**Fig. 2:**
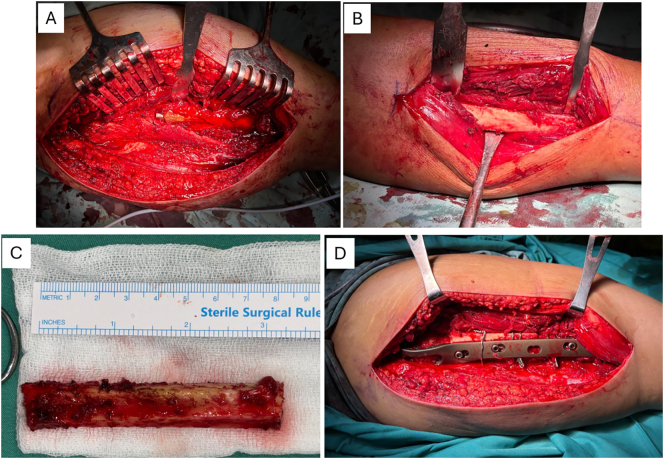
Intraoperative procedure. (A) Comminutive fracture at right proximal femur, (B) preparation for fibular graft harvest, (C) 8 cm fibular graft was harvested, (D) fixation with trocantheric claw plate, fibular strut graft insertion, and cable-wiring.

The patient was planned for implantation of plate and screw with fibular strut graft. The lateral incision of femur was made exposing the fracture site. A trochanteric claw plate was then positioned over the fracture site. Fixation was achieved using three screws in the proximal fracture fragment and two screws in the distal cortical segment of the femoral shaft. An 8 cm fibular graft was obtained from the same leg, and was split into two parts. The graft then placed anteriorly and posteriorly to the fracture site. Graft fixation was performed using three cable wires to ensure stability and alignment (Fig. 2). The wound was sutured in layered fashion.

**Fig. 3:**
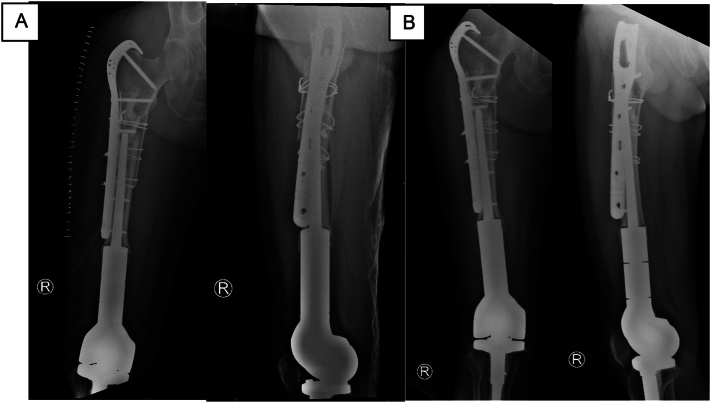
Post-operative radiograph. (A) 1-day post operative, (B) 6-weeks post operative.

The patient was able to partially bear weight with two crutches after 6 weeks, then with one crutches after 12 weeks. The 6-week follow-up X-ray evaluation showed a good fixation result (Fig. 3). The patient was able to walk with no assistance after four months. The modified Harris hip score was assessed to give a comprehensive and objective evaluation of the pain, gait, and functional activity. Occasionally, pain was still complained by the patient; but his activity was not negatively affected by the pain. Regarding his gait, although he has a slight limp, he was able to walk without limitation. Moreover, he was able to climb the stairs normally with a banister. He had no difficulty wearing socks or shoes, was able to sit in any chair for around more than 1 h, and was unable to utilize public vehicles as a mode of transportation. A good range of motion was observed during follow-up clinical assessment; however, this was based on subjective evaluation without formal goniometric measurement or standardized functional scoring beyond the modified Harris hip score. In summary, the modified Harris hip score was 81, interpreted as a good result.

## 3 Discussion

Periprosthetic fractures following megaprosthesis reconstruction are rare but potentially devastating, with incidence estimates from 2% to 10% depending on tumor type, implant location, and follow-up duration [14]. In Indonesia, epidemiological data on megaprosthesis utilization and associated complications remain limited. Reports from tertiary centers in Jakarta and Surakarta have reported only small case series, reflecting both the rarity and the specialized nature of this procedure [5,6]. Consequently, every documented case provides valuable insights for surgical decision-making and postoperative outcomes in resource-constrained environments.

Management of periprosthetic fractures in patients with a megaprosthesis is challenging due to altered anatomy, limited bone stock, and compromised biology. Treatment options include revision megaprosthesis, allograft-prosthetic composite reconstruction, internal fixation with plates and grafts, and in extreme cases, amputation [16,17]. The selection of strategy hinges on the fracture location, implant stability, bone quality, and patient factors such as age, function, and oncologic status. In cases where the implant remains stable and sufficient bone exists for fixation, internal fixation with a plate-augmented with biological material—is a favored limb-salvage approach [14,15].

Across periprosthetic femoral fractures with a stable stem, internal fixation using locking plates augmented with cortical strut grafts shows consistently high union. Series of periprosthetic femoral fractures treated with plate strut allograft report union rates approximately 92–98% with time-to-union at 6–18 months, as struts act as biologic plates that enhance construct stiffness and the healing surface [18,19]. Meta-analytic data across surgically managed periprosthetic femoral fractures still identify nonunion and infection as leading failure modes, reinforcing the value of biologic augmentation when fixation is feasible [20].

In this case, the periprosthetic femur fracture likely resulted from localized bone fragility induced by prior bone cement insertion at the exact fracture site. Cementation, while essential for implant fixation in megaprosthesis, can alter the local bone microenvironment by increasing thermal necrosis risk and reducing remodeling capacity, especially at the cement–bone interface. Over time, this can weaken the surrounding cortical bone, making it more susceptible to fracture under minimal trauma or physiological stress.^**17**^ The fracture pattern observed aligns with this mechanism, emphasizing the importance of monitoring cemented zones for potential late complications.

A trochanteric claw plate was selected due to the fracture's proximity to the proximal femoral segment, which includes the greater trochanter. Trochanteric claw plates are specifically designed to grip the greater trochanter and offer rotational control, making them suitable for managing periprosthetic fractures around the femoral stem, particularly when the stem is well-fixed and a revision is not warranted. Furthermore, they allow secure fixation without disrupting the prosthetic component, preserving limb length and alignment [21].

To enhance biomechanical stability and biological healing, a fibular strut graft was placed anteriorly and posteriorly to the fracture site. The fibula provides cortical strength, acting as a biological plate, and has the potential to integrate with host bone, contributing to long-term fracture union. Autologous fibular grafts are especially useful in oncologic patients with compromised healing potential, and in cases where implant-based fixation may be insufficient alone [22,23]. This case is unique in demonstrating the use of a trochanteric claw plate combined with a fibular strut graft to treat a periprosthetic femoral fracture around a stable megaprosthesis. Rather than opting for a complex revision, the treatment strategy was carefully considered based on the limited residual bone stock, rendering conventional fixation methods alone inadequate. Biological augmentation using a fibular strut graft not only enhances mechanical stability by acting as a bridging scaffold but also promotes osteoconduction and integration at the fracture site. The graft serves to offload stress from the fixation construct and expands the surface area for healing in compromised bone [23,24]. To the best of our knowledge, there are few reports documenting this precise construct for megaprosthetic periprosthetic fractures, making this case both innovative and clinically valuable.

Functional outcome was assessed using the Modified Harris Hip Score (mHHS), a validated tool commonly used to evaluate hip function, pain, and daily activity performance, particularly in reconstructive hip surgery. Although originally designed for total hip arthroplasty, the mHHS remains a relevant and practical metric in the assessment of lower limb function following limb salvage procedures [24]. The patient of this case achieved an mHHS of 81 at four months postoperatively, indicating a good functional result. This reflects satisfactory pain control, ambulatory capacity, and overall hip function, supporting the viability of using claw plate fixation combined with fibular strut grafting as a limb-preserving strategy in selected patients with stable megaprostheses.

The main limitation of this case report is the relatively short follow-up period of four months. While the initial outcome, including fracture stabilization and functional recovery, appears favorable, this duration is insufficient to assess critical long-term endpoints such as complete fracture union, implant durability, or the risk of late complications including aseptic loosening or local tumor recurrence. We propose structured surveillance: (1) clinical and radiographic reviews at 6, 12, and 24 months (standing AP/lateral femur; CT if union is equivocal); (2) serial functional outcomes (mHHS, gait status); (3) infection surveillance (exam, inflammatory markers if symptomatic); and (4) oncologic follow-up per tumor protocol. Documenting radiographic union and freedom from reoperation ≥12 months will contextualize this construct against published union and complication benchmarks for plate-and-strut fixation around stable stems and megaprostheses.

## 4 Conclusion

This case highlights a limb-salvage approach for a periprosthetic femoral fracture after megaprosthesis in a giant cell tumor, using a trochanteric claw plate, cable wire, and fibular strut graft. The primary challenge was the severely limited residual bone stock, which restricted options for secure fixation and increased the risk of mechanical failure. The combination of mechanical fixation and biological augmentation provided sufficient stability and support, allowing implant preservation and early mobilization. The patient achieved a good short-term outcome with a Modified Harris Hip Score (mHHS) of 81. This technique offers a viable alternative to revision surgery, particularly in settings with limited resources or implant availability. Further studies with larger patient cohorts and longer follow-up are warranted to validate the durability and effectiveness of this approach.

## Author contribution

Muhamad Naseh Sajadi Budi: Surgeon, Conceptualization, Methodology, Investigation, Writing - Original Draft; Supervision

Bangkit Primayudha: Operating Assistant, Conceptualization, Methodology, Investigation, Writing - Original Draft; Supervision

Mohammad Syarif Mas'ud: Data Curation, Formal Analysis, Visualization, Writing – Review & Editing

Greesea Dinamaria Whitiana: Methodology; Investigation; Writing – Original Draft

Andre Prawiradinata: Resources; Project Administration; Validation; Writing – Review & Editing

## Consent

The patient's parents/legal guardian received an explanation of the procedures and possible risks of the surgery and gave written informed consent. Our manuscript does not contain any individual person data. Written informed consent was obtained from the patient's parents/legal guardian for publication and any accompanying images. A copy of the written consent is available for review by the Editor-in-Chief of this journal on request. No video was taken.

## Ethical approval

Ethical approval was provided by the Ethical Committee of Medical Faculty, Padjadjaran University, Bandung, Indonesia.

## Guarantor

The guarantor in this study is Muhamad Naseh Sajadi Budi.

## Research registration number

This study was approved by the Ethical Committee of the Faculty of Medicine, Padjadjaran University (LB.03.11/X.1.9/621/2024).

## Funding

This research did not receive any specific grant from funding agencies in the public, commercial, or not-for-profit sectors.

## Conflict of interest statement

The authors declare that there is no conflict of interest regarding the publication of this paper. The following case has never been presented at any conferences or scientific meetings.
